# Engineered procyanidin-Fe nanoparticle alleviates intestinal inflammation through scavenging ROS and altering gut microbiome in colitis mice

**DOI:** 10.3389/fchem.2023.1089775

**Published:** 2023-03-29

**Authors:** Yongliang Chang, Xiawei Wu, Shengwei Lu, Jiahao Du, Yixiu Long, Yefei Zhu, Huanlong Qin

**Affiliations:** ^1^ Shanghai Clinical College, Anhui Medical University, Shanghai, China; ^2^ The Fifth Clinical Medical College of Anhui Medical University, Hefei, China; ^3^ Department of General Surgery, School of Medicine, Shanghai Tenth People’s Hospital Affiliated to Tongji University, Shanghai, China; ^4^ Medical School of Nantong University, Nantong, China; ^5^ Department of Gynecological Oncology, Fudan University Shanghai Cancer Center, Fudan University, Shanghai, China; ^6^ Department of Oncology, Shanghai Medical College, Fudan University, Shanghai, China

**Keywords:** inflammatory bowel disease (IBD), procyanidins, Pc-Fe nanoparticles (Pc-Fe), reactive oxygen species (ROS), gut microbiome

## Abstract

Inflammatory bowel disease (IBD) is an idiopathic chronic inflammatory bowel disease characterized by inflammation, intestinal barrier injury, and imbalance of gut microbiota. Excess accumulation of reactive oxygen species (ROS) is closely correlated with the development and reoccurrence of IBD. Previous researches demonstrate that procyanidin, as a natural antioxidant, exhibits strong ability of eliminating ROS, thus showing good therapeutic effects in the inflammation-related diseases. Non-etheless, its poor stability and solubility always limits the therapeutic outcomes. Here, we typically designed an antioxidant coordination polymer nanoparticle using the engineering of procyanidin (Pc) and free iron (Fe), named Pc-Fe nanozyme, for effectively scavenging ROS and further inhibiting inflammation while altering the gut microbiome for the treatment of colitis. Furthermore, *in vitro* experiments uncover that Pc-Fe nanoparticles exert strong multi biomimic activities, including peroxidase, and glutathione peroxidase, for the scavenging of ROS and protecting cells from oxidative injury. In addition, the colon accumulation of Pc-Fe nanozyme effectively protects the intestinal mucosa from oxidative damage while significantly downregulates pro-inflammatory factors, repairs the intestinal barriers and alternates gut microbiome after orally administrated in sodium dextran sulfate (DSS) induced colitis mice. The results collectively illustrate that the multienzyme mimicking Pc-Fe nanozyme owns high potential for treating IBD through scavenging ROS, inhibiting inflammation, repairing gut barriers and alternating gut microbiome, which further promising its clinical translation on IBD treatment and other ROS induced intestinal diseases.

## 1 Introduction

Chronic relapsing-remitting inflammatory bowel disease (IBD) is characterized by bloody diarrhea and chronic pain, weight loss, malabsorption of water and nutrients (MacEachern et al., 2015; Schuster et al., 2015). Due to its high prevalence rates, recurrence and intractability, IBD has become a major economic burden and public health issue globally (Sudabeh Alatab et al., 2020). Furthermore, patients with IBD have an increasingly higher risk of developing colorectal cancer (CRC), because chronic inflammation drove neoplastic progression, and many molecular similarities have been found between colitis-associated CRC and sporadic CRC (Zahary et al., 2012). Recent studies have also demonstrated the roles of the microbiome consistent with the host immune system in the progress of colitis and colitis associated colorectal cancer (CAC) (Cao P et al., 2019). While 5-aminosalicylic acid (5-ASA), steroids and immunosuppressive drugs are widely utilized in clinical practice for IBD treatment (Ungaro et al., 2019), their clinical outcomes are greatly limited by side effects such as the nausea, diarrhea, abdominal pain, moon face, buffalo waist etc (Löwenberg and D'Haens, 2013; Mendell et al., 2020; Toffoli et al., 2017). Therefore, the boost of effective and safe drugs for IBD is urgently needed.

Recent studies have shown a strong correlation between inflammatory diseases and reactive oxygen species (ROS) (Fleming et al., 2019; Hsu et al., 2022; Zhou et al., 2022). In the process of acute inflammatory diseases, such as IBD, the accumulation of ROS exacerbates localized tissue injury, leading to long-lasting chronic inflammation, which increases the risk of cancer (Liu et al., 2020). Additionally, abnormally high ROS levels cause oxidative stress to both host cells and the gut microbiome (Hall et al., 2017), though neutrophils kill the pathogenic bacteria *via* the generation of ROS (Jorgensen et al., 2017). Furthermore, trimethylamine-N-oxide, a choline-derived metabolite produced by the gut microbiome, increases ROS production in a dose and time-dependent manner by inhibiting ATG16L1 and LC3- II (Yue et al., 2017). Active ingredients in traditional Chinese medicine, such as polyphenols, alkaloids, quinones, and terpenoids, alleviate IBD through a multi-target mechanism with rare adverse reactions (Cao S Y et al., 2019). As one of the natural phenols, procyanidin is widely distributed in various traditional Chinese medicinal materials (Baselga-Escudero et al., 2014), while exhibiting strong antioxidant and free radical elimination abilities, relieving edema, improving hypoxia and defending radiation (Bak et al., 2016; Han et al., 2019; Chen et al., 2021; Yan et al., 2022). Therefore, procyanidin has become a research hotspot in inflammatory diseases related to ROS. For instance, procyanidin promoted cell autophagy, activated Nrf2 signaling pathway and inhibited activation of NLRP3 inflammasome, inhibiting ROS accumulation, thus can be utilized in the treatment of fatty liver, Parkinson’s Disease and renal damage (Yang et al., 2011; Cao P et al., 2019; Chen et al., 2021). Besides, procyanidins B2 could repair damaged gut barriers and inhibit colitis-associated tumorigenesis *via* the suppression of oxidative stress in colitis mice (Zhu et al., 2021). What’s more, Chen et al. (2017) found that procyanidin ameliorates experimental colitis in mice *via* reducing ROS signaling in macrophages. The reduced ROS signaling downregulates MMP9 expression, thereby suppress NF-κB signaling and interrupt the formation of the NLRP3 inflammasome (Chen et al., 2017). To sum up, though the procyanidin has promising therapeutic potentials for IBD treatment, its clinical applications is always limited for the instability and poor solubility.

**FIGURE 1:**
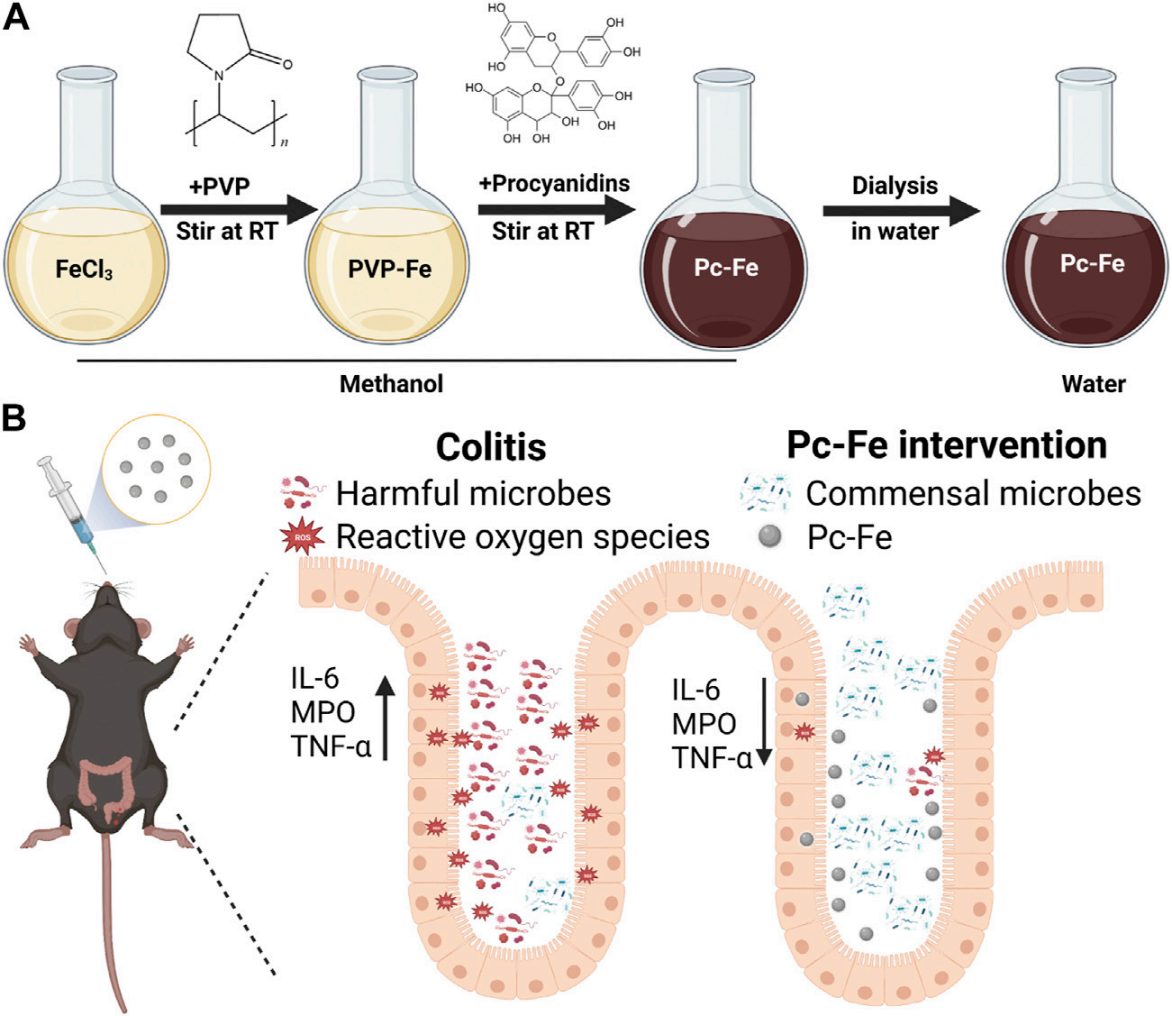
**(A)** The synthesis procedure of Pc-Fe and **(B)** schematic illustration of IBD relief with Pc-Fe. **(B)** During the process of acute inflammation (left part), the levels of ROS increased and harmful bacteria may attach to the injured intestinal epithelium, which inhibit commensal microbes to colonize and make function and destroy the gut barrier by up-regulating pro-inflammatory factors. After Pc-Fe intervention (right part), the levels of ROS decreased and probiotics Acidimicrobiia increased, which alleviated the progression of acute intestinal inflammation through down-regulating the pro-inflammatory factors, and expelling potential pathogenic bacteria.

Excitingly, stable complexes can be formed between metal ions and HO–C groups of natural antioxidants including curcumin, quercetin and gallic acid, which therefore improves their water solubility and stability. Additionally, the increased stability and solubility of these antioxidants enhance their efficiency in removing free radicals from tissues and reducing inflammation as well (Zhang et al., 2021). Hence, we designed a kind of engineering coordination polymer nanoparticles (Pc-Fe NPs) formed by procyanidin and free iron (Fe) ions *via* the connection of coordinate covalent bond (Figure 1A). Compare with procyanidin, Pc-Fe exhibited ideal stability and solubility, which further improving the bioavailability. The results of our study confirm the excellent antioxidative ability and multi-biomimic activity of Pc-Fe NPs. Besides, Pc-Fe NPs enhances cellular resistance to H_2_O_2_-induced oxidative stress. Remarkably, we observe that the symptoms of IBD are significantly ameliorated and abnormally alleviated colon inflammation returned to normal after orally gavage of Pc-Fe on DSS-induced colitis mice (Figure 1B). In addition, Pc-Fe also reshapes the gut microbiome and may exert the potential benefits for the IBD treatment. According to our study, coordination polymer nanoparticles based on natural antioxidant products and metal ions have a promising future in the clinical translation on the treatment of IBD and other ROS related intestinal diseases.

## 2 Experimental section

### 2.1 Materials

Procyanidin (MW 594.52, P823328) was purchased from Shanghai Macklin Biochemical Co., Ltd. (Shanghai, China). Dextran sulfate sodium salt (DSS, MW 36,000–50,000 Da, 216011090) was purchased from MP Biomedicals. The dialysis bag (3500 Da, MD3544) was provided by Shanghai Yuanye Bio-Technology Co., Ltd. (Shanghai, China). Iron chloride hexahydrate (FeCl_3_•6H_2_O, MW 270.3, F102739) and 5-Aminosalicylic acid (MW 168.15, A129982) were purchased from Shanghai Aladdin Biochemical Technology Co., Ltd. Polyvinylpyrrolidone (PVP, MW 29,000, PVP 360) and methanol were purchased from Sigma-Aldrich (St. Louis, Mo, United States). The CCK-8 cell viability assay kit (CCK8, C0039) was obtained from Beyotime Biotechnology (Shanghai, China).

Thirty healthy male 4-week-old C57BL/6J mice (20 ± 2 g) were purchased from Huachuang Sino Medical Technology Co., Ltd. (Jiangsu, China). The mice were bred and maintained under specific pathogen-free (SPF) conditions, and all mouse procedures were conducted according to institutional animal care guidelines. The Shanghai Tenth People’s Hospital Laboratory Animal Ethics Committee approved this study (License no. SHDSYY-2022-KY3206).

### 2.2 Methods

#### 2.2.1 Preparation of Pc-Fe nanoparticles

Preparation of Pc-Fe was performed according to a previously reported method (Zhang et al., 2021). Polyvinylpyrrolidone (PVP) (66 mg) was dissolved in 5 mL of methanol solution. FeCl_3_•6H_2_O (20 mg) was dissolved in 1 mL of methanol. Procyanidin (10 mg) was dissolved in 1 mL methanol. The methanol solution of FeCl_3_•6H_2_O was added drop by drop to the methanol solution of PVP, and the mixture was stirred at a speed of 300 rpm for 5 min. The methanol solution of procyanidin was then added drop by drop to the solutions containing iron and PVP, and the mixture was stirred at a speed of 300 rpm for 3 h at room temperature. To obtain the final product, the above synthetic products were dialyzed in pure water for 12 h.

#### 2.2.2 Characterizations of Pc-Fe

The morphology and elemental composition of Pc-Fe were observed using a transmission electron microscope (FEI Talos F200X, Thermo Fisher Scientific, United States). The changes in absorption spectra during the preparation of Pc-Fe were evaluated using a UV/Vis spectrophotometer (UV-2600i, SHIMADZU, Japan). The Pc-Fe was analyzed by a Fourier-transform infrared (FT-IR) instrument (Thermo Scientific Nicolet iS20, United States), with a range of 400–4,000 cm^−1^. The hydrodynamic size and zeta potential of the Pc-Fe were measured by ZSU3100 (Malvern Instruments, Malvern, United Kingdom). X-ray photoelectron spectroscopy (XPS) spectra of Pc-Fe were obtained using Thermo Scientific’s K-Alpha (United States). Thermogravimetry analysis (TGA) is done from 28.68 to 600 °C, with a heating rate of 10 °C on PerkinElmer STA 8000 (PerkinElmer, United States).

#### 2.2.3 Stability of Pc-Fe at light condition

The stability of the Pc-Fe at light condition was evaluated by a UV/Vis spectrophotometer (UV-2600i, SHIMADZU, Japan) and ZSU3100 (Malvern Instruments, Malvern, United Kingdom). Pc-Fe was dispersed in pure water. UV/Vis absorption spectra of the sample solutions were recorded at 0, 24, 48, 72, 96, and 120 h. Hydrodynamic sizes of Pc-Fe in pure water, phosphate buffer saline (PBS), Dulbecco’s modified Eagle’s medium (DMEM) and fetal bovine serum, were recorded at 0, 24, 48, 72, 96, and 120 h.

#### 2.2.4 OH scavenging activity of Pc-Fe

An OH scavenging activity test was conducted using the TMB chromogenic method. In the Fenton reaction, H_2_O_2_ and Fe^2+^ combine to generate oxidized TMB (oxTMB) with a characteristic absorption at 650 nm. Therefore, the concentration of remaining OH can be determined *via* monitoring the absorption at 650  nm of oxTMB. The working test solutions were divided into three groups which contained 250  μM TMB, 1  mM FeSO_4_; 250  μM TMB, 10  mM H_2_O_2_, 1  mM FeSO_4_; and 250  μM TMB, 10  mM H_2_O_2_, 1  mM FeSO_4_ and Pc-Fe respectively. The preparation took place in the dark and resting took place for 15 min. Afterward, the color changes of the solutions were observed and the absorbance peaks at 650  nm of solutions at 15, 30, 45, and 60 min were monitored with a UV-vis spectrophotometer.

#### 2.2.5 Cell culture

NCM460 cell line (the colon normal epithelial cell line, BFN608006385) and Caco2 cell line (the human colonic carcinoma cell line, BFN60800651) were obtained from the Chinese Academy of Sciences’ Cell Bank. We cultured NCM460 and Caco2 in DMEM supplemented with 10% FBS and 1% penicillin-streptomycin at 37°C in a 5% CO_2_ incubator.

#### 2.2.6 Cell safety evaluation and antioxidant activity

Cell safety evaluation and antioxidant activity of Pc-Fe were tested on NCM460 and Caco2 cell lines. For safety evaluation, we seeded 5,000 cells per well in 96-well plates and incubated them overnight to ensure cell attachment. The cell culture medium was replaced with fresh media containing various concentrations of Pc-Fe (0, 0.001, 0.01, 0.1, 1, 10, 100 and 1,000 μg/mL), and incubated for 24 h. Using a microplate reader (SpectraMax iD5, Molecular Devices, United States), absorbance was measured at 450 nm (reference = 650 nm) to determine cell viability. Pc-Fe antioxidant activity was tested in 96-well plates by seeding cells at 5,000 cells per well and incubated overnight. The medium was replaced by fresh medium containing various concentrations of Pc-Fe (0, 0.1, 1, 10, and 100 μg/mL) before incubated for 24 h. Then H_2_O_2_ and Fe^2+^ were added to well with final concentrations of 200 μM and 40 μM, respectively, and incubated for 24 h. Cell viability was measured. In these experiments, untreated cells and medium served as controls. Data are expressed as mean ± SEM.

#### 2.2.7 Animals experiments

A total of 20 mice were divided into four groups randomly (5 mice in each group), namely, PBS, DSS + PBS, DSS+5-ASA and DSS + Pc-Fe. Acute colitis mice models were established by adding 3% Dextran Sulfate Sodium (DSS) to the drinking water for 7 days, except for the PBS group (Birchenough et al., 2016). The PBS group and DSS + PBS group were given orally 200 μL PBS. 200 μL 5-ASA (100 mg/kg) or 200 μL Pc-Fe (100 mg/kg) per mouse per day were orally administered to candidates in the DSS+5-ASA group or DSS + Pc-Fe group respectively. Each day, the mice’s physiological status and body weight were recorded. Fecal and anal status were observed, and the disease activity index (DAI) was calculated. On the 7th day, the mice were sacrificed and dissected. We collected feces and colon sections from each group of mice, and measured the colon length in each one.

#### 2.2.8 *In vivo* toxicity assessment

Ten male C57BL/6J mice (20 ± 2 g) were divided into two groups randomly, including the PBS group and the Pc-Fe group (five mice per group). The mice were orally gavaged with 200 μL of PBS or of Pc-Fe (100 mg/kg) per mouse per day for seven consecutive days and their weights were recorded daily. On the 7th day, their blood was collected and delivered for blood biochemical evaluations after the mice were sacrificed. Additionally, the major organs, including the heart, liver, spleen, lung, and kidney were collected and fixed for histopathological staining.

#### 2.2.9 Histopathological examination

The colon tissues were fixed in a 4% paraformaldehyde solution after sacrificing the mice and paraffin-embedded sections were stained with H&E and Masson’s trichrome to determine tissue morphology. Based on previously published methods, a board-certified pathologist blinded examined cecal samples to determine pathology scores (Zhang et al., 2015). Evaluation criteria were as follows: Epithelium (E): 0, normal morphology, 1, goblet cell loss, 2, goblet cell loss in large areas, 3, loss of crypts, 4, loss of crypts in large areas. The infiltration (I) score is (I): 0, no infiltration; 1, infiltration around crypt base; 2, infiltration related to the L. muscularis mucosae; 3, extensive infiltration observed both in the L. muscularis and thickening of the mucosa; 4, whole L. submucosa infiltration. The total histological score is defined as the sum of the epithelium and infiltration score (total score = E + I) (Obermeier et al., 1999). The activities of IL-6, MPO and TNF-α were measured by immunohistochemistry. Immunohistochemistry assays in colon tissues were performed following the method reported previously (Khare et al., 2014). Immunohistochemistry analysis was done on paraffin-embedded mouse intestine. Serial tissue sections (4 μm) Swiss rolls were stained for IL-6 (Abcam, United States, ref#ab233706), MPO (Abcam, United States, ref#ab208670) and TNF-α (Abcam, United States, ref#ab270264) using standard procedures. We dried slides, de-waxed them in xylol, and then rehydrated them using decreasing alcohol concentrations. A 15% H_2_O_2_ solution in methanol was used to block endogenous peroxidase, followed by 10 mM citrate buffer. After that, a 0.2% horse serum, 3% BSA solution in TRIS buffer was used to block slides. The anti-IL-6, MPO, TNF-α and biotinylated goat antibodies were incubated overnight at 4°C, followed by the avidin-biotin-HRP complex. Using DAB, the staining was visualized and hematoxylin was used to counterstain the nuclei. Histofluid was used to embed dehydrated slides. Pathologists blindly examined cecal sections from a board-certified pathologist to count the IL-6, MPO, and TNF positive cells.

#### 2.2.10 Gut microbiota 16S rDNA sequencing assay

We prepared fecal samples according to the manufacturer’s instructions and extracted the DNA from them as previously stated (Kong et al., 2021; Zhu et al., 2022). We examine DNA samples using a Nanodrop 2000 UVvis spectrophotometer (Thermo Scientific, Wilmington, MA, United States) and 1% agarose gel electrophoresis. Amplicon PCR system was used to amplify 16S rDNA’s V3 + V4 region using barcode specific primers. In order to detect PCR products, 2% agarose gel electrophoresis was used, followed by gelation and recovery using an AxyPrep DNA gel recovery kit (Axygen Biosciences, Union City, CA, United States). The primer sequence was 341F:5′- CCTACGGGNGGCWGCAG-3’; 806R: 5′-GGACTACHVGGGTATCTAAT-3’. DNA samples were quantified and homogenized, then sequenced using Illumina NovaSeq PE250 (San Diego, CA, United States). R (v3.5.1) was used for statistical analysis.

#### 2.2.11 Statistical analysis

The mean +SEM was used to express the data. A statistical analysis was conducted using the GraphPad Prism 8.0 software (CA, United States). The significance of differences among three or more groups was assessed using a one-way ANOVA analysis. Statistical significance was indicated by *p < 0.05, **p < 0.01, and ***p < 0.001.

## 3 Results and discussion

### 3.1 Synthesis and characterizations of Pc-Fe nanoparticles

**FIGURE 2:**
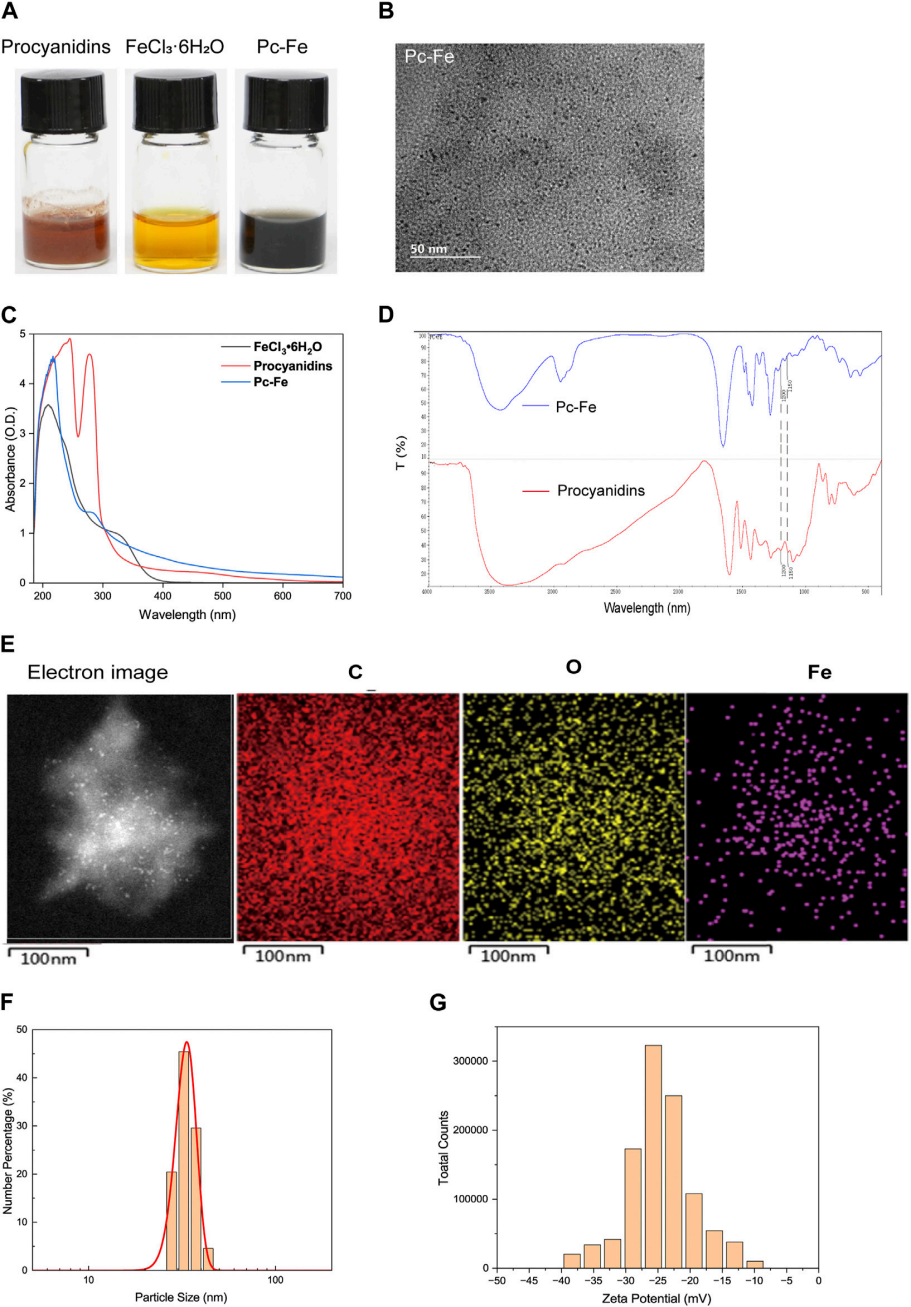
Synthesis and characterizations of Pc-Fe. **(A)** Physical drawing of Pc-Fe at room temperature for 7 days. **(B)** TEM images of Pc-Fe. **(C)** The UV-vis absorbance spectra of FeCl_3_•6H_2_O, procyanidins and Pc-Fe. **(D)** FTIR spectra of procyanidins and Pc-Fe samples. **(E)** EDS element mapping analysis of C, O, and Fe in Pc-Fe. **(F)** Size distribution of Pc-Fe in pure water. **(G)** Zeta potential of Pc-Fe.

**FIGURE 3:**
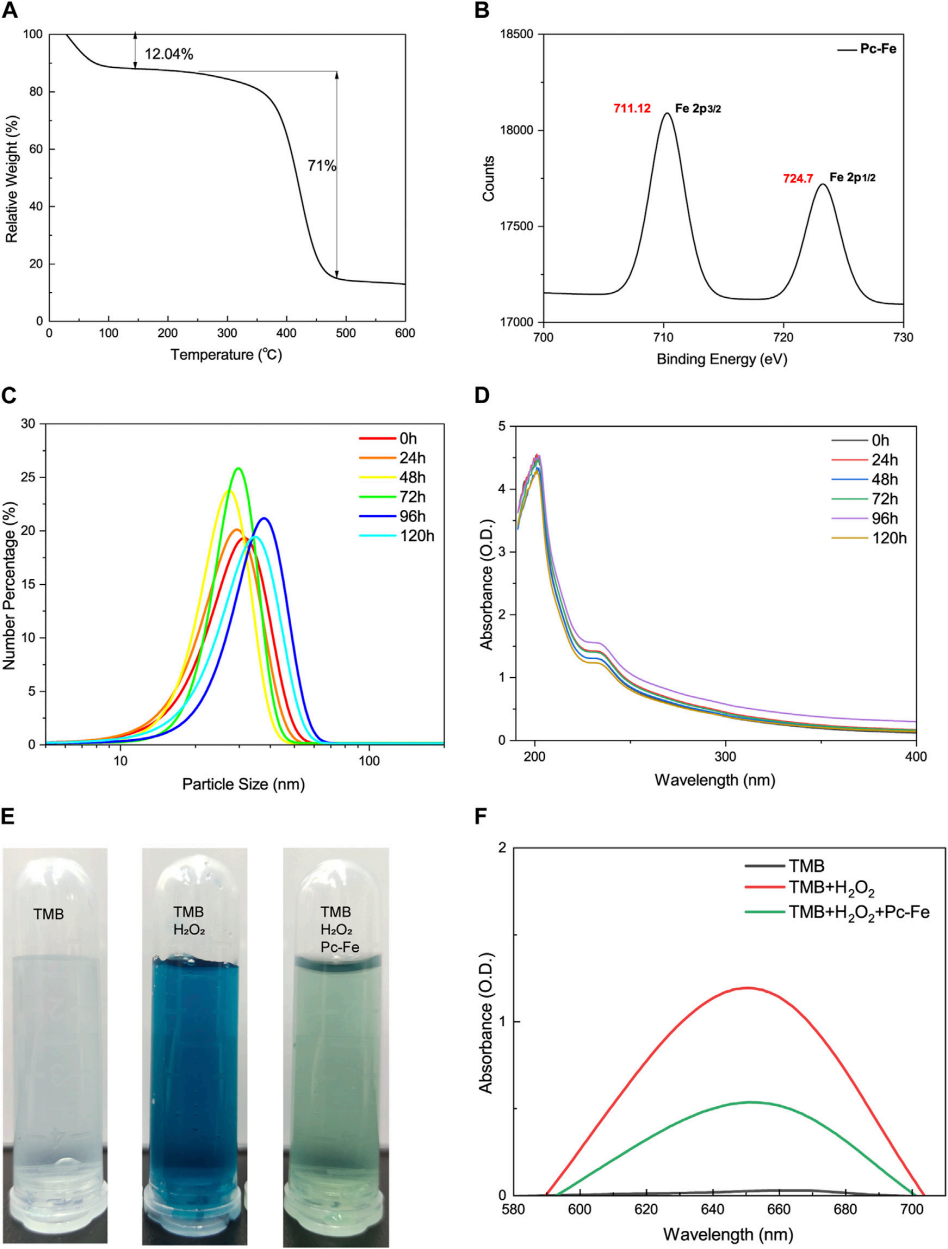
Characterizations of Pc-Fe. **(A)** Thermogravimetric analysis of Pc-Fe. **(B)** XPS spectra of Fe in Pc-Fe. **(C)** Size distributions of Pc-Fe in pure water at 0, 24, 48, 72, 96, and 120 h. **(D)** The UV-vis absorbance spectra Pc-Fe at 0, 24, 48, 72, 96, and 120 h. **(E)** The TMB chromogenic results of Pc-Fe. TMB is colorless and transparent; TMB + H_2_O_2_ is dark blue; TMB + H_2_O_2_+Pc-Fe is pale blue. **(F)** The UV-vis absorbance spectra of TMB with Pc-Fe.

The schematic of the three-step fabrication process for obtaining the Pc-Fe nanoparticles was shown in Figure 1A. Ferric ions were added to procyanidin methanol solutions to form Pc-Fe nanoparticles before PVP was added to this solution in order to facilitate nanoparticle growth and improve their dispersion in water according to previous report (Gong et al., 2016). The procyanidin phenol groups were successfully coordinated with ferric ions in the solution, evidenced by that its color changed from brownness to deep dark (Figure 2A). To remove the excessive ferric ions, the solution was dialyzed against pure water overnight. The synthesized nanoparticles were then transferred to pure water. After being kept at room temperature for 7 days, Pc-Fe still dispersed well in water, indicating the good solubility and stability (Figure 2A). We further explored the physical properties of Pc-Fe. The size and morphology of nanoparticles were shown in the TEM image, the size of Pc-Fe was about 12 nm (Figure 2B). According to UV/Vis absorption spectra result, the characteristic absorption peak of Pc-Fe nanoparticles appeared at 220 nm, which was different with the counterparts in FeCl_3_•6H_2_O or procyanidin alone, indicating the successful preparation of Pc-Fe nanoparticles (Figure 2C) (Shen et al., 2020). In the FTIR spectra of Pc-Fe, the decreased infrared intensity at 1,150–1,200 cm^−1^ (HO–C stretching band) indicated that Fe^3+^ and HO–C groups of procyanidin were successfully coordinated (Figure 2D) (Zhang et al., 2021). The corresponding element mapping showed that Pc-Fe contained the elements of C, O, and Fe, which further witnessed the successful preparation of Pc-Fe (Figure 2E). Dynamic light scattering (DLS) results convinced that Pc-Fe had a hydrodynamic diameter of about 25 nm, slightly larger than the size observed through TEM results (Figure 2F). Such differences might due to the minor difference between size distribution data obtained by DLS and the results estimated *via* the TEM images (Chen et al., 2015). The American Society of Testing Materials (ASTM) has declared that the zeta potential is closely related to the degree of dispersion and stability of materials, and the zeta potential measure is an effective evaluative method for material stability (Yin et al., 2013). Therefore, the zeta potential of Pc-Fe was tested and the surficial zeta potential for Pc-Fe was about −25 mV, endowing the nanodot good water dispersibility **(** Figure 2G). Encouraged by the successful synthesis of Pc-Fe NPs, the molecular composition of Pc-Fe NPs was further identified. Based on thermogravimetry analysis, the molar ratio of procyanidin to ferric ions was 3:1 (Figure 3A). The XPS spectrum revealed that the 711 eV and 724 eV binding energy peaks can be attributed to Fe 2p3/2 and 2p1/2 of Fe (III), respectively, indicating that ferric ions are in a stable oxidative state (Zhang et al., 2021) (Figure 3B). As the major composition of Pc-Fe, poor light stability restricts the application of procyanidin, so the light stability of Pc-Fe was tested. Figures 3C, D showed the rare change for the size distributions and UV/Vis absorption spectra of Pc-Fe from 0 to 120 h, suggesting the good light stability of Pc-Fe. The peroxidase (POD) mimicking ability of PC-Fe was then investigated and the TMB chromogenic method was used to test the OH scavenging activity of Pc-Fe. As a neutral substance, TMB can be oxidized to the blue ox-TMB in the presence of peroxide, such as H_2_O_2_ and ROS generated by the reaction of Fe^2+^ and H_2_O_2_ (Liu et al., 2018). During the same time, compared to dark blue solution containing TMB, Fe^2+^ and H_2_O_2_, the color of solution consisting of TMB, Fe^2+^, H_2_O_2_ and Pc-Fe, was pale blue, indicating that Pc-Fe has excellent reactive oxygen species scavenging ability (Figure 3E). Accordingly, the special absorbance peak of ox-TMB is at 650 nm, and the value at 650 nm of solution consisting TMB, Fe^2+^, H_2_O_2_ and Pc-Fe was lower than that of solution containing TMB, Fe^2+^ and H_2_O_2_, which further convinced the results above (Figure 3F). Based on the results mentioned before, we confirmed that Pc-Fe nanozyme synthesized was a kind of tiny and stable nanoparticles with antioxidant capacity.

### 3.2 *In vitro* safety evaluation and antioxidant activity studies

**FIGURE 4:**
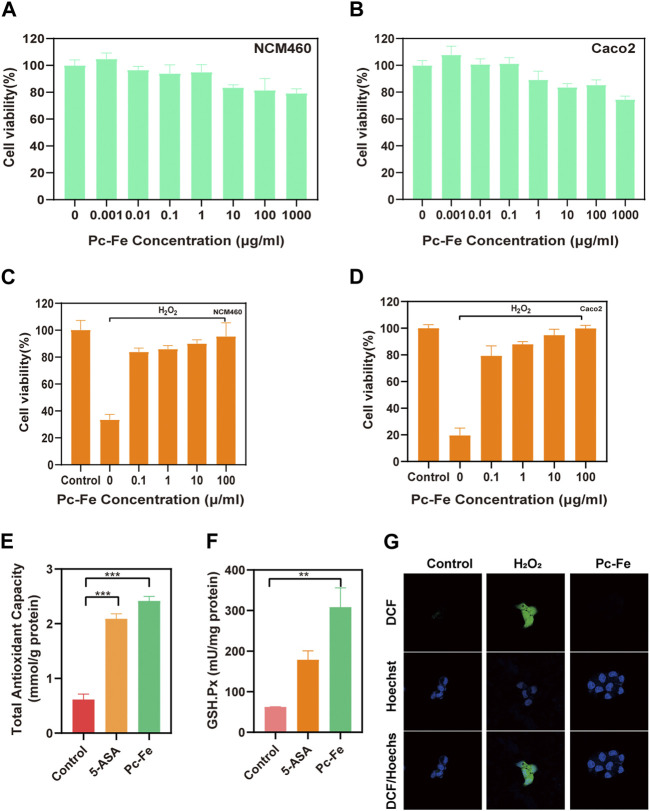
Cell safety evaluation and antioxidant activity of Pc-Fe. **(A,B)** Cell viability of NCM460 and Caco2 cells after Pc-Fe incubation. **(C,D)** Cell viability of NCM460 and CaCo_2_ cells under H_2_O_2_ with Pc-Fe. **(E,F)** The total antioxidant capacity and cellular glutathione peroxidase (GPx) of cell with Pc-Fe. **(G)** Confocal microscope images of Pc-Fe for eliminating ROS.

We further explored the cell safety and antioxidant activity of Pc-Fe. The cytotoxicity of the Pc-Fe nanoparticles was tested on NCM460 and Caco2 cell lines *via* cell counting kit-8 (CCK-8) assay. Both cell lines did not show significant changes in cell viability after exposure to Pc-Fe at the tested concentrations demonstrating the lack of cytotoxic effects (Figures 4A, B). Then, the antioxidant activity of Pc-Fe in NCM460 and Caco2 cell lines was measured. Due to the oxidative damage induced by H_2_O_2_, the cell viabilities in both cell lines decreased after incubated with H_2_O_2_ at 200 μM for 4 h. However, such tendency was reversed by the addition of Pc-Fe under non-cytotoxic conditions, and the protect effect of Pc-Fe was strengthen with the increasing concentration of Pc-Fe added (Figures 4C, D), indicating that Pc-Fe effectively protected colon epithelial cells from ROS, induced injures, and such effect was increased with the increasing of concentration. We hypothesized such benefits might largely owned to the multienzyme mimicking activities of Pc-Fe nanoparticles. Thus, the intracellular level of total antioxidant capacity and glutathione peroxidase (GPx) were further investigated after the Pc-Fe or 5-ASA treatment. As shown in Figure 4E, Pc-Fe observably upregulated the intracellular total antioxidant capacity compared to control group or 5-ASA group. Additionally, compared with the control group or 5-ASA, Pc-Fe significantly increased intracellular GPx (Figure 4F). Such results convinced that the total antioxidant capacity and GPx mimicking ability of Pc-Fe nanoparticles was stronger than 5-ASA, and could explain the protection effect for oxidative damage, which was in correlation with previous research (Turk et al., 2017). In addition, the anti-ROS ability of Pc-Fe was estimated *in vitro* by laser scanning confocal microscopy (LSCM). 2,7-dichlorodihydrofluorescein diacetate (DCFH-DA) immunofluorescence staining was used to evaluate the intracellular ROS scavenging capability of Pc-Fe. DCFH was hydrolyzed from DCFH-DA by the intracellular esterase, before DCF that emitting green fluorescence intensity was generated by the reaction of intracellular ROS and DCFH (Chen H et al., 2019). Thus, the green DCF fluorescence intensity was in line with the intracellular ROS level. The appearance of strong green intensity in H_2_O_2_ group demonstrated the excess ROS generation after the co-incubation of 200 μM H_2_O_2_ for 4 h in Caco2 cells, which was in line with previous reports. While rare green intensity was observed in both control group and Pc-Fe group, indicating that the Pc-Fe treatment significantly reduced the intracellular (Figure 4G). To sum up, the *in vitro* studies further confirmed the Pc-Fe NPs protected cells from the excessive oxidative damage, mainly through its multienzyme mimicking capacity, as well as the good cellular biocompatibility of Pc-Fe.

### 3.3 Therapeutic effects of Pc-Fe nanoparticles in DSS induced colitis mice

**FIGURE 5:**
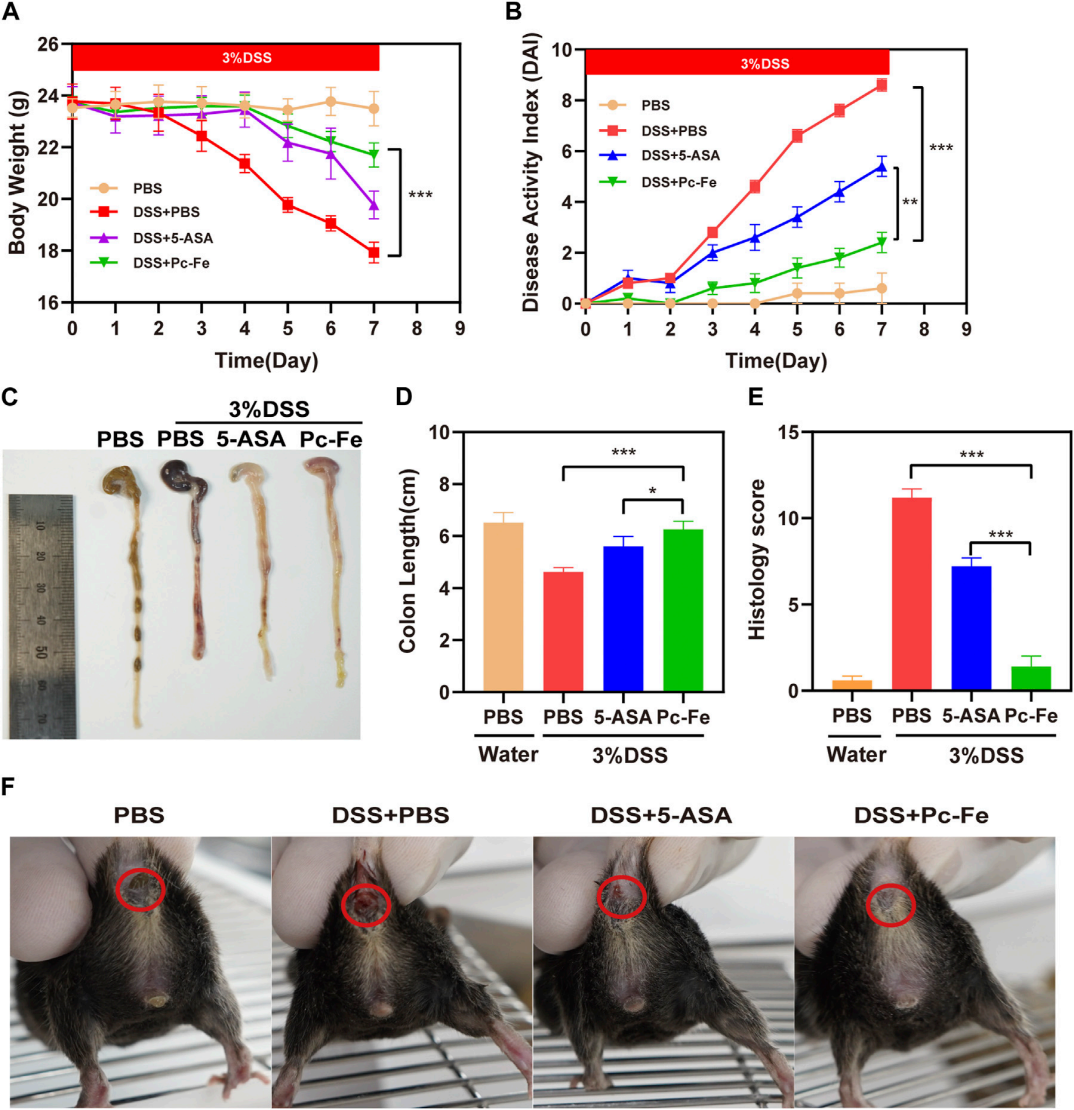
Pc-Fe alleviates UC in the DSS-induced colitis model. **(A)** Body weight of mice in each group for 7 days. **(B)** The DAI score in each group. **(C)** Representative images of the colon in the PBS group, the DSS + PBS group, the DSS+5-ASA group and the DSS + Pc-Fe group on 7th day. **(D)** Colon length shown as a chart. **(E)** The HDI scores in each group based on inflammatory cell infiltration and mucosal damage under microscopic observation. TMB is colorless and transparent; TMB + H_2_O_2_ is dark blue; TMB + H_2_O_2_+Pc-Fe is pale blue. **(F)** Representative images of the anus in the PBS group, the DSS + PBS group, the DSS+5-ASA group and the DSS + Pc-Fe group on 7th day. . One-way ANOVA test was utilized, and data were shown as mean ± SEM. *p < 0.05; **p < 0.01; ***p < 0.001; ****p < 0.0001.

Encouraged by the *in vitro* results, we then evaluated the therapeutic effect in colitis mice. The DSS-induced colitis model is broadly used in researches of IBD (Czarnewski et al., 2019). Colitis mice model were established using 3% DSS dissolved in drinking water (Chassaing et al., 2014; Wang et al., 2020). Weight, fecal character and general condition are main parameters used for evaluation of enteritis severity (Ozkul et al., 2020). Mice treated with PBS showed normal behavior (feeding, grooming, and social interactions) with smooth hair, normal fecal characteristics, stable body weight gaining and a DAI index nearing zero. However, the DSS + PBS mice tended to maintain a hunched position, developing sparse hair growth, and decreasing food and water intake, indicating the successful establishment of colitis mice model. Previous report convinced that mice exposed to DSS developed colitis with decreased body weights, increased disease active index (DAI), and shorten colons (Hwang et al., 2016), thus we further analyzed the changes of body weight, DAI index and colon length to fully evaluate the clinical outcomes of 5-ASA and Pc-Fe treatment. As shown in Figure 5A, the body weights of mice in all groups remained stable during the first 3 days and began to decrease from the fourth day. On the fourth day, significant weight loss was observed in both the DSS + PBS group and DSS+5-ASA group. Differently, no obvious weight loss was observed in the DSS + Pc-Fe group (*p* < 0.001) (Figure 5A), indicating that Pc-Fe exceeded 5-ASA in inhibiting the weight loss in colitis mice. Furthermore, Further, the severity of colitis was evaluated by DAI scores of all mice as previous described (Ge et al., 2018). The DAI index of the DSS + PBS group remained stable during the first 2 days, while increased from the third day, and remained constant for the following 4 days. Compared with the DSS + PBS group, the DAI index of the DSS+5-ASA group kept at a relatively low level and was significantly decreased on the seventh day (*p* < 0.01), which was consistent with previous reports (Zhu et al., 2022). Moreover, significantly decreased DAI index was observed in DSS + Pc-Fe group comparing to DSS + PBS group and DSS+5-ASA group, which indicated the Pc-Fe surpassed 5-ASA for the alleviation of the colitis activation, demonstrating the stronger therapeutic outcomes of Pc-Fe for treating colitis comparing to 5-ASA (Figure 5B). The changes of colon length, therefore, reflected the severity of colitis and the therapeutic efficacy (Jackson et al., 2018). As shown in Figures 5C, D, the colon length was significantly shortened after DSS treatment, which could be remarkably revised by the Pc-Fe (*p* < 0.001). Meanwhile, compared to DSS+5-ASA, the colon length of the DSS + Pc-Fe group was longer (*p* < 0.05). In addition, from the fifth day, mice in the DSS + PBS group presented with diarrhea and hematochezia and mice in the DSS+5-ASA group showed minor diarrhea and hematochezia, while no obvious sign of diarrhea or hematochezia was observed in the DSS + Pc-Fe group mice (Figure 5F). According to these results, we illustrated that Pc-Fe significantly alleviated the symbols of DSS-induced colitis, and its therapeutic efficiency was obviously better than that of 5-ASA.

### 3.4 The effects of Pc-Fe nanoparticles on colon pathological characteristics in DSS-induced colitis

**FIGURE 6:**
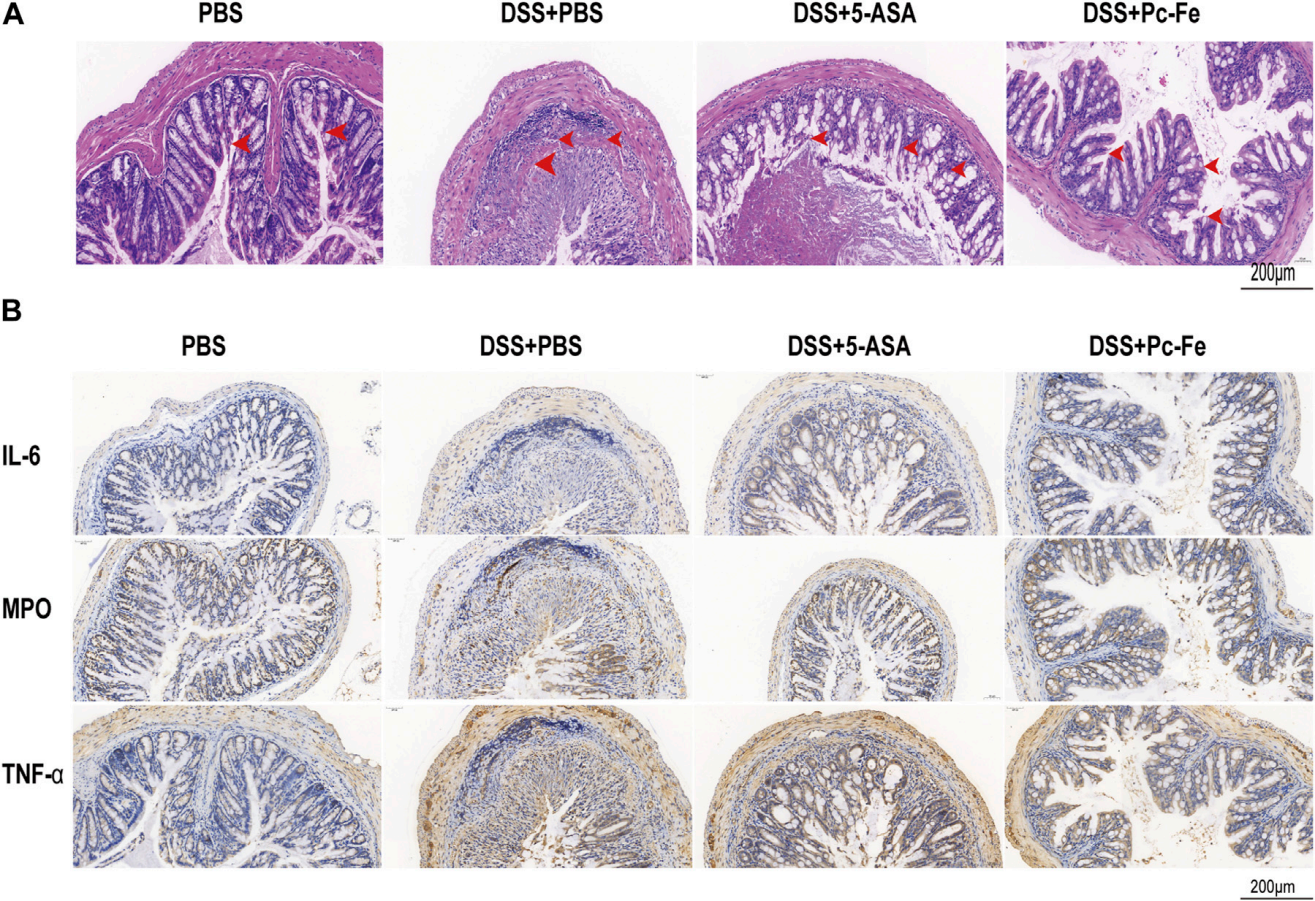
Histopathological examination of Pc-Fe. **(A)** Representative image of colon tissue stained with H&E from each group. **(B)** Immunofluorescence staining image of mice. Sections of tissue were stained with IL-6 and MPO and TNF-α from each group.

The intestinal barrier structure and the severity of colonic inflammation of mice were further evaluated *via* H&E staining and immunohistochemistry (IHC) staining, respectively (Chen Q et al., 2019). Compared to the PBS group, tissue section in the DSS + PBS group showed significantly mucosal erosion, ulceration, occurrence, inflammatory cell infiltrations, and loss of crypts (red arrows represent inflammatory cells infiltrations, and crypt loss) (Figure 6A), indicating the sever disease activity in control group. Meanwhile, though no obvious inflammatory cell infiltration was seen, there still remained mucosal erosions and inflammatory cells infiltrations in the colon section of DSS+5-ASA group (red arrows showed mucosal erosion), indicating the relief of colitis. The DSS + Pc-Fe group had few inflammatory cell invasions compared to the DSS + PBS group, demonstrating that Pc-Fe ameliorated inflammatory cell infiltration of colitis. Besides, in the DSS + Pc-Fe group, the lumen and secretions were clearly visible, and no obvious shedding or disordered arrangement of tissue cells was seen, demonstrating the potential benefits of Pc-Fe for repairing gut barriers. To better understand the disease activity of each group, the histopathology scores were analyzed. And the result showed that the histological score of the DSS + Pc-Fe group was significantly lower than the counterparts in DSS + PBS group ((*p* < 0.001) or DSS+5-ASA group (*p* < 0.001), showing the therapeutic effect of Pc-Fe was better than 5-ASA (Figure 5E). Recent studies showed that excess accumulated ROS activated inflammatory transcription pathways and increased the production of pro-inflammatory factors (Donato et al., 2018), which in turn initiated the production of excess ROS (Alharthi et al., 2020). Therefore, we typically evaluated the expression of IL-6, MPO and TNF-α, which were reported to be the pro-inflammatory factors and the inflammation statement indicators for colitis, in colon tissue sections (Saleh et al., 2019; Singh et al., 2019). As shown in Figure 6B, the DSS + Pc-Fe group showed significantly lower IL-6, MPO and TNF-α expressions compared to the DSS + PBS group and 5-ASA group, suggesting that Pc-Fe could remarkably inhibit the inflammation statement in DSS-induced colitis. As a result, ROS-scavenging Pc-Fe nanozymes alleviated the DSS induced colitis, decreased the levels of proinflammatory factors levels, and potentially improved the gut barriers.

### 3.5 Safety evaluation of Pc-Fe

**FIGURE 7:**
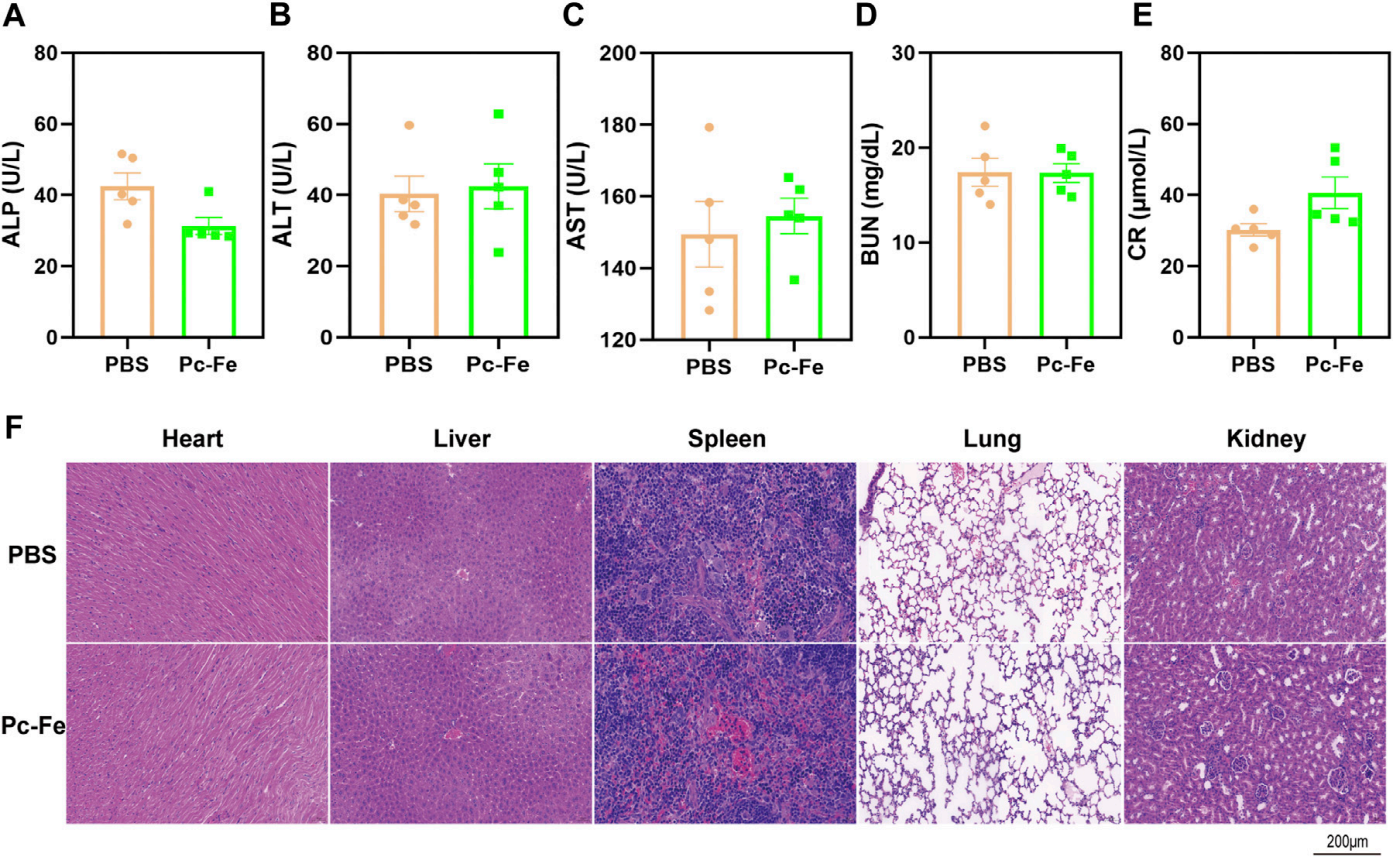
*In vivo* toxicity assessment of Pc-Fe. **(A–E)** Blood hematological analysis of mice after oral administration with Pc-Fe. (Data is shown as mean ± SEM). **(F)** H&E staining of the sections of major organs including heart, liver, spleen, lung and kidney harvested from the mice in the PBS group and the Pc-Fe group.

The safety of Pc-Fe was evaluated by blood biochemistry and histopathological examinations. Liver and kidney are the major organs that regulate absorption, distribution, metabolism and excretion of drugs (Sprowl et al., 2016). After 7 days’ gavage of PBS or Pc-Fe, the mice were sacrificed and a serious of biochemical parameters of liver or kidney, including blood urea nitrogen, creatinine, alkaline phosphatase, glutamic-pyruvic transaminase and aspartate aminotransferase were tested. The liver function markers (alkaline phosphatase, glutamic-pyruvic transaminase and aspartate aminotransferase) and kidney function markers (creatinine and blood urea nitrogen) in the Pc-Fe treated mice were within the normal range, showing no significant difference with those in the PBS treated group (Figures 7A–E). Additionally, compared to PBS group, no obvious adverse effects on the tissue sections of major organs, including heart, liver, spleen, lung, and kidney were observed in mice treated with Pc-Fe (Figure 7F). Thus, nanoparticles were considered safe for C57BL/6J mice.

### 3.6 The regulatory effect of Pc-Fe on the gut microbiome

**FIGURE 8:**
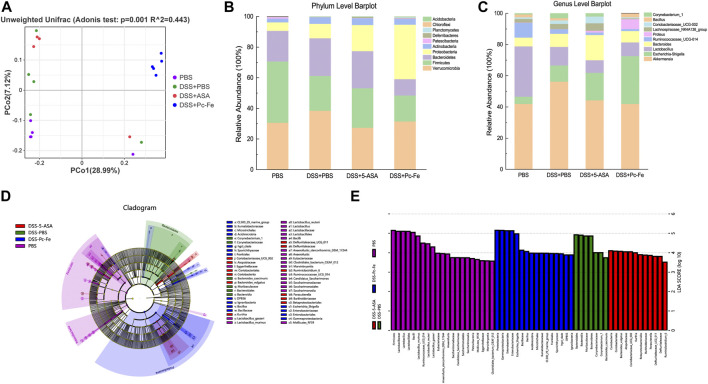
Diversity and composition of gut microbiota. **(A)** Weighted Unifrac PCoA analysis was utilized to distinguish bacterial clustering. The component proportion of the microbiome at the phylum **(B)** and genus level **(C)** among groups was shown. The relative abundance of genera difference was identified *via* the Wilcoxon rank-sum test. The phylogenetic distribution in the PBS group, the DSS + PBS group, the DSS+5-ASA group and the DSS + Pc-Fe group was illustrated with a cladogram **(D)** and a histogram **(E)**.

Gut microbiome play an important role in the progress of IBD (Ananthakrishnan et al., 2017). So, the 16S rDNA sequence of the gut microbiome was used to investigate the gut microbiome alternation after different treatments. According to the principal coordinate (PCA) analysis based on weighted UniFrac distance, the composition of gut microbiota differed significantly among the groups (Figure 8A). *Bacteroidetes* were more abundant in DSS + PBS group at the phylum level, but decreased in the DSS + Pc-Fe group (PBS: 20%, DSS + PBS: 24.6%, DSS+5-ASA: 24.26%, DSS + Pc-Fe: 10.66%), while that of *Proteobacteria* was significantly increased in the DSS + Pc-Fe group (PBS: 5.56%, DSS + PBS: 9.48%, DSS+5-ASA: 17.06%, DSS + Pc-Fe: 35.59%) (Figure 8B). Previous studies revealed that *Bacteroidetes* display higher relative abundance in the colonic mucosae of UC patients (Lucke et al., 2006). Additionally, compared to the PBS group *Firmicutes* was less abundant in DSS + PBS group, DSS+5-ASA group and DSS + Pc-Fe group (PBS:40.05%, DSS + PBS: 22.71%, DSS+5-ASA: 25.78%, DSS + Pc-Fe: 17%). At the genus level, a higher relative abundance of *Bacillus* and *Corynebacterium-1* was observed in the DSS + PBS group, while a decreased tendency was observed in the DSS + Pc-Fe group (PBS: 41.98%, DSS + PBS: 56.3%, DSS+5-ASA: 44.34%, DSS + Pc-Fe: 10.66%; PBS: 0.69%, DSS + PBS: 3.04%, DSS+5-ASA: 1.94%, DSS + Pc-Fe: 0.01%, individually). Meanwhile, *Lactobacillus* abundance was decreased in the DSS + PBS group, DSS+5-ASA group and DSS + Pc-Fe group as compared with the PBS group (PBS: 32.26%, DSS + PBS: 11.93%, DSS+5-ASA: 8.06%, DSS + Pc-Fe: 8.75%) (Figure 8C). In total, Pc-Fe reshaped the gut microbiome of DSS-induced IBD mice. Further, using LEfSe analysis, the different groups were analyzed to determine their key elements. *Bacillus*, which has been reported to inhibit inflammation and enhance gut barrier function, was significantly reduced by DSS treatment at the genus level. Notably, previous research demonstrated that the abundance of *Bacillus* was related to the alleviation of colitis (Zhu et al., 2022), which potentially illustrated the underlying mechanisms for Pc-Fe alternating gut microbe and alleviating colitis. Moreover, the major composition of the microbiome in the DSS + Pc-Fe group were, *Bacillus*, *Acidimicrobiia*, *Gammaproteobacteria*, *Enterobacteriales*, and *Frankiales*, in contrast with the dominant bacteria in the DSS+5-ASA group were *Coriobacteriales*, *Bacteroides-vulgatus*, Atopobiaceae and *Kurthia*. (Figures 8D, E). These results suggested that Pc-Fe reshaped the gut microbiome of DSS induced colitis mice by increasing the relative abundance of *Bacillus*, *Proteobacteria*, and *Acidimicrobiia*, which therefore potentially relieved intestinal inflammation and promoted intestinal function recovery.

In summary, given the poor stability and solubility of procyanidin, we prepared Pc-Fe in a simple and cost-effective way, making it favorable for future practical applications. With small nanoparticle size, Pc-Fe exhibited excellent stability and solubility compared to procyanidin. Pc-Fe retains the ability to eliminate ROS from procyanidin, thus downregulating the levels of pro-inflammatory cytokines. Importantly, the synergistic effectiveness of Pc-Fe in simultaneously eliminating ROS and modulating microbiota homeostasis in the colonic microenvironment provided significant therapeutic efficacy against DSS-induced IBD. But the potential mechanisms of how Pc-Fe treat IBD need further exploration. Additionally, the safety of Pc-Fe needs further evaluation before clinical applications, and the therapeutic effects need to be tested in other diseases associated with inflammation.

## 4 Conclusion

In this study, a stable Pc-Fe nanozymes mimicking the activities of POD and GPx for the development of anti-oxidative system that could be utilized in the elimination of various ROS was proposed and engineered. A serious of *in vitro* investigations further observed that Pc-Fe performed ideal cellular protecting effect against oxidative damages, typically, the ROS scavenging and multienzyme mimicking capacity. *In vivo* studies also discovered that with high biocompatibility, Fc-Pe nanozyme not only alleviated the symptoms and improved the gut barrier function, but also achieved the inflammation inhibition and the anti-oxidative damage protecting. Further investigations identified that the inflammation regulation and gut microbiome regulation severed as the potential mechanism for the admirable therapeutic outcomes of Pc-Fe nanozymes on DSS-induced colitis models, especially in the aspect of pro-inflammatory cytokine downregulation, reducing inflammatory cell infiltration, and reshaping the gut microbiome dysbiosis. Therefore, the engineering Pc-Fe nanozyme we synthesized possessing the excellent ROS scavenging ability, advanced inflammation inhibition performance, admirable gut microbiome alternation effect, and ideal biocompatibility could function as an emerging antioxidant drug with high therapeutic performance for colitis treatment, and other oxidative damage related intestinal diseases.

## Data Availability

The datasets presented in this study can be found in online repositories. The names of the repository/repositories and accession number(s) can be found below: https://www.ncbi.nlm.nih.gov/- PRJNA900208.
