# Supplementary material for: Engineered procyanidin-Fe nanoparticle alleviates intestinal inflammation through scavenging ROS and altering gut microbiome in colitis mice
Source: Front Chem. 2023 Mar 29;11:1089775. doi: 10.3389/fchem.2023.1089775 (PMC10090317; doi:10.3389/fchem.2023.1089775)
Supplement: Supplementary file 1 [file DataSheet1.PDF]

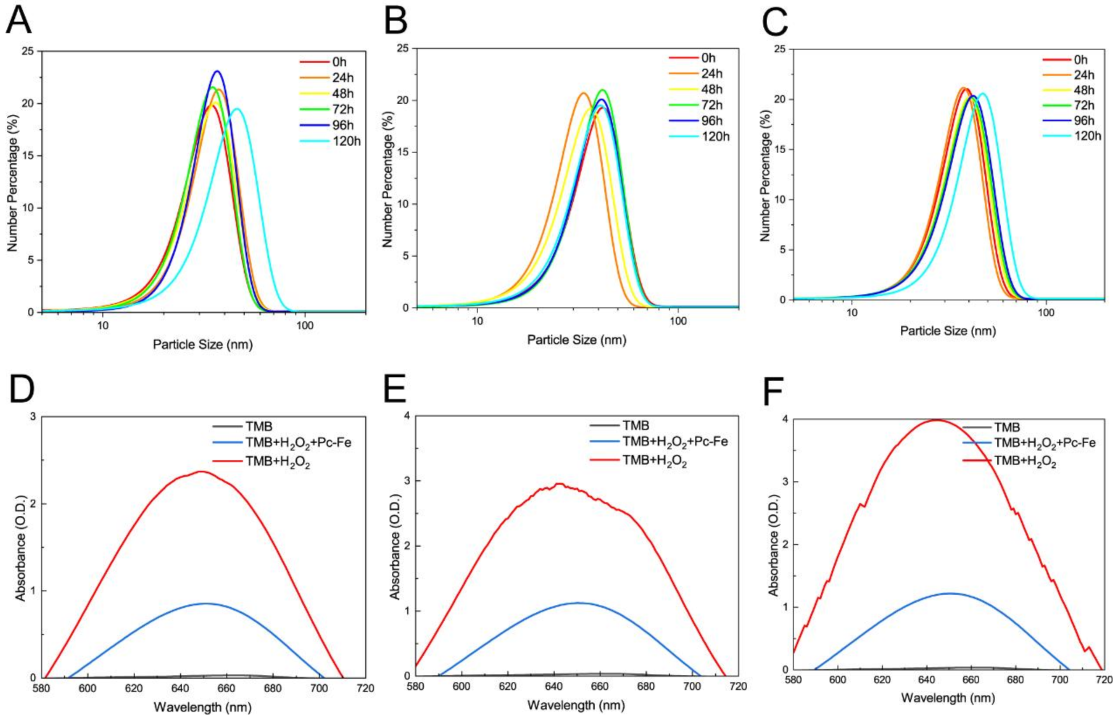

Figure S1. Stability and ·OH scavenging activity of Pc-Fe. Size distributions of Pc-Fe in PBS (A), DMEM (B) and FBS (C) at 0, 24, 48, 72, 96 and 120h. The absorbance peak in 652 nm of the solutions which contain 250 μM TMB, 1 mM FeSO4; 250 μM TMB, 10 mM H2O2, 1 mM FeSO4; and 250 μM TMB, 10 mM H2O2, 1 mM FeSO4 and Pc-Fe respectively at 30 (D), 45 (E) and 60 (F) minutes.
